# Cost-effectiveness of risk stratified medication management for reducing premature cardiovascular mortality in Kenya

**DOI:** 10.1371/journal.pone.0218256

**Published:** 2019-06-25

**Authors:** Sujha Subramanian, Rainer Hilscher, Robai Gakunga, Breda Munoz, Elijah Ogola

**Affiliations:** 1 RTI International, Waltham, Massachusetts, United States of America; 2 University of Nairobi and Kenyatta National Hospital, Nairobi, Kenya; Heidelberg University, GERMANY

## Abstract

**Background:**

Cardiovascular disease (CVD) is a major contributor to the burden from non-communicable diseases in Sub-Saharan Africa and hypertension is the leading risk factor for CVD. The objective of this modeling study is to assess the cost-effectiveness of a risk stratified approach to medication management in Kenya in order to achieve adequate blood pressure control to reduce CVD events.

**Methods:**

We developed a microsimulation model to evaluate CVD risk over the lifetime of a cohort of individuals. Risk groups were assigned utilizing modified Framingham study distributions based on individual level risk factors from the Kenya STEPwise survey which collected details on blood pressure, blood glucose, tobacco and alcohol use and cholesterol levels. We stratified individuals into 4 risk groups: very low, low, moderate and high risk. Mortality could occur due to acute CVD events, subsequent future events (for individual who survive the initial event) and other causes. We present cost and DALYs gained due to medication management for men and women 25 to 69 years.

**Results:**

Treating high risk individuals only was generally more cost-effective that treating high and moderate risk individuals. At the anticipated base levels of effectiveness, medication management was only cost-effective under the low cost scenario. The incremental cost per DALY gained with low cost ranged from $1,505 to $3,608, which is well under $4,785 (3 times GPD per capita) threshold for Kenya. Under the low cost scenario, even lower levels of effectiveness of medication management are likely to be cost-effective for high-risk men and women.

**Conclusions:**

In Kenya, our results indicate that the risk stratified approach to treating hypertension may be cost-effective especially for men and women at a high risk for CVD events, but these results are highly sensitive to the cost of medications. Medication management would require significant financial investment and therefore other interventions, including lifestyle changes, should be evaluated especially for those with elevated blood pressure and overall 10-year risk that is less than 20%.

## Introduction

Cardiovascular diseases (CVD) are the leading cause of mortality worldwide and were responsible for more than 17 million deaths in 2015. [1] Of these deaths, 7.4 million people died of coronary heart disease and 6.7 million from strokes. [1] CVDs account for over a third of the premature deaths globally. [2] More than three quarters of the CVD deaths occur in low- and middle- income countries and this poses a substantial burden in terms of premature mortality in this setting. Hypertension, or high blood pressure, is the largest contributor worldwide to CVD events but other risk factors such as tobacco use, harmful use of alcohol, diabetes and hyperlipidaemia also increase the risk of CVD events and related mortality. [2,3]

Kenya is one example of a middle-income country with high rates of hypertension and other CVD risk factors. [4–6] Data from the 2015 Kenya STEPwise survey on non-communicable diseases estimated that 24% of adults aged 18 to 69 years had raised blood pressure, defined as systolic blood pressure (SBP) ≥ 140 and/or diastolic blood pressure (DBP) ≥ 90 mmHg. [7] The prevalence of high blood pressure among young adults 18 to 29 years was 13% which clearly highlights the early onset of hypertension among Kenyans. [7] Although the average prevalence was similar between men and women, there were some gender differences with women experiencing lower rates than men during young adulthood and higher rates than men in their 50s and 60s. Severe hypertension, which is defined as SBP ≥160 mm Hg and/or DBP ≥100 mm Hg, affected about 8% of the adult population surveyed. [7]

Importantly, in addition to hypertension, Kenyans also have a high prevalence of other underlying CVD risk factors. The STEPwise survey data [7] showed that about 25% of men 45 to 69 years and 19% of men 18 to 44 years were smokers. Almost a quarter of men in these same age groups were also moderate or heavy alcohol users. Women, on the other hand, were generally not smokers nor heavy drinkers but 21% of women 45 to 69 years and 12% of those 18 to 44 years were classified as obese. Overall, among those aged 18 to 44 years, about 10% had three or more risk factors for cardiovascular disease, while among those aged 45 to 69 years, approximately 26% had three or more risk factors.

Scientific studies have consistently provided evidence that lowering blood pressure through pharmacological and behavioral interventions can reduce disability and mortality. [8–10] For example, randomized control trials have reported that a reduction in SBP of 10 mmHg is associated with a 22% reduction in coronary heart disease and 41% reduction in stroke, [11] Historically, the Kenyan guideline for management of hypertension had recommended treatment only for cases with severe hypertension but more recent guidance from the Ministry of Health has expanded the use of medication management to those with SBP ≥ 140 and/or diastolic blood pressure (DBP) ≥ 90 mmHg. [12,13] Medication management is estimated to be extremely low in Kenya; in 2015, less than 5% of those with high blood pressure were on hypertension medications. [7]

In this study, we explore the optimal target cohort for medication management to control high blood pressure and reduce CVD risk and mortality in Kenya. We use a risk stratified approach to identify those who are most likely to benefit from physician guided use of hypertension medications. As a substantial proportion of the Kenya population has multiple risk factors that contribute to CVD mortality, we use both raised blood pressure as well as overall risk of CVD events to triage individuals into risk-based groupings. The findings from this study will provide a systematic evaluation of the benefits and cost of treating patients based on the underlying risk of developing CVD events and offer guidance for the implementation of evidence-based policies to reduce CVD mortality and morbidity.

## Materials and methods

### Overall approach

**Fig 1:**
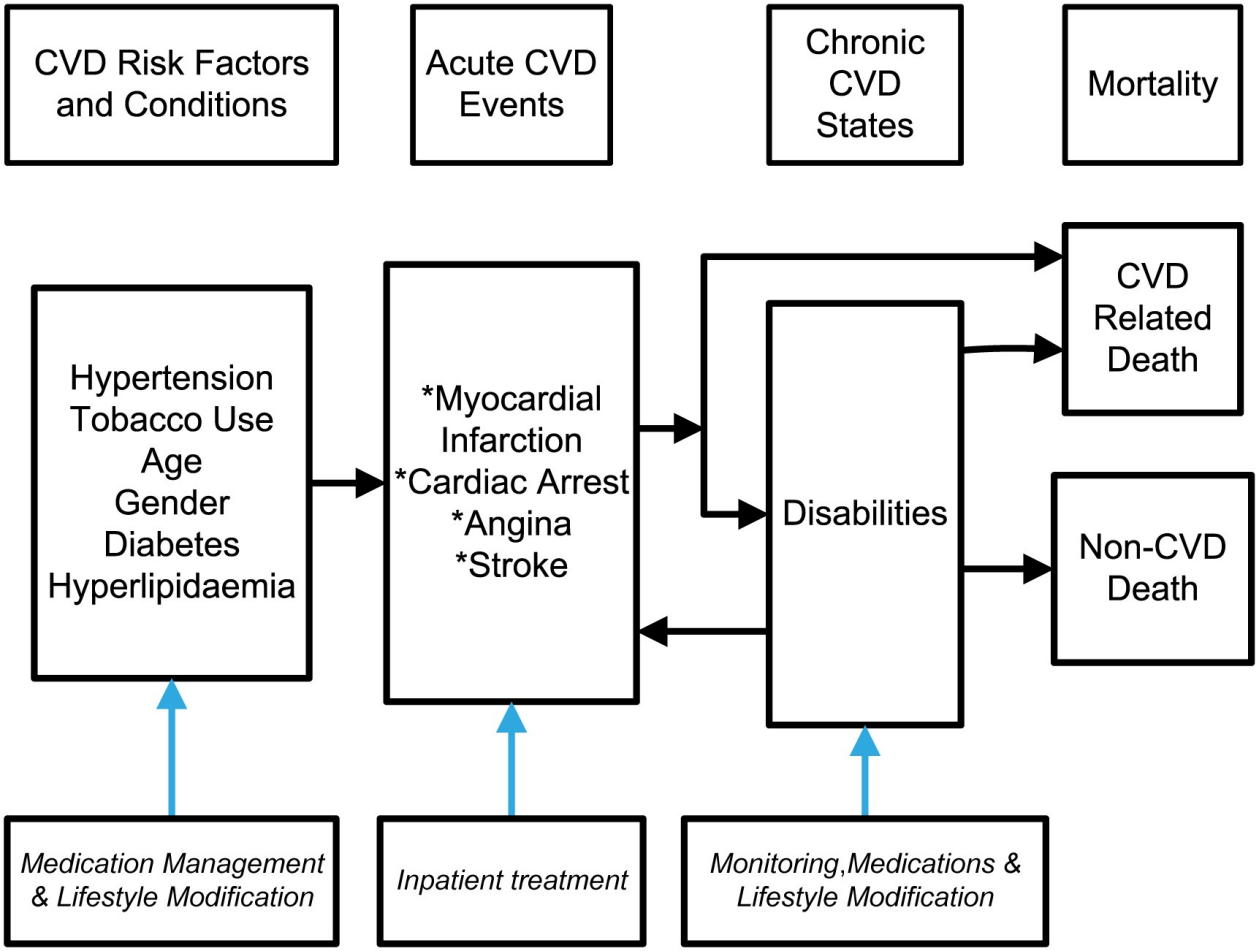
Framework for modeling CVD events and mortality.

We developed a simulation model using risk profiles from the Kenyan population to generate CVD events, mortality and cost to assess the benefits and cost-effectiveness of implementing medication management for individuals at higher than average risk of experiencing CVD events. Fig 1 presents the framework used to develop our modeling approach. We began with the risk factors for CVD which included hypertension (both SBP and DBP values), tobacco use, age, gender, diagnosis of diabetes and cholesterol levels (hyperlipidemia). These risk factors are used to derive risk scores to predict the probability of events related to cardiovascular heart disease (CHD) (cardiac arrest, myocardial infarction [MI] or angina) and stroke. These events could lead to CVD death, either immediately within the first year or in future years. Individuals who survive the acute CVD event are modeled to experience disabilities due to the CVD events. We also include death from other causes based on age-specific mortality rates. We calculated the probability of a non-CVD death using the life tables provided by the World Health Organization (WHO). [14]

### Risk stratification

Data on risk factors to calculate the risk of CVD events were obtained from the Kenya STEPwise survey on non-communicable diseases. [7] This 2015 STEPwise survey is the first nationally representative survey that provides comprehensive information on risk factors for NCDs, including CVD events. A total of 4,500 eligible individuals aged 18 to 69 years were successfully interviewed, representing a high response rate of 95%. Of these, 99% consented to anthropometric measurements of blood pressure and heart rate, height, weight, and waist and hip circumference. Biochemical measurements of fasting blood glucose, triglyceride, and cholesterol was also collected among a further 93% who provided consent. The survey provides detailed person level data to derive CVD risk scores that are representative of the Kenyan population. We applied the risk profiles previously reported from the Framingham study [15] to generate annual person level CVD event risk based on age, gender, SBP, DBP, smoking status, presence or absence of diabetes and cholesterol levels. We categorized each of these scores into 5-year age groupings to stabilize our estimates because of the small sample size available for each specific age group. As shown in Table 1, we assigned individuals to four risk levels using the SBP, DBP and 10-year CVD risk for each age group. Separate categories were developed for women and men based on gender-based risk profiles (See Tables A and B in S1 File for risk values). Individuals could move to higher risk categories as they aged in our model as age is a key independent risk factor for CVD. The risk of a first stroke or CHD event was derived for each risk category and age group based on separate Framingham risk profiles [16,17]. Probability of stoke and CHD risk events were first derived in 5-year age increments for each risk category and then annual probabilities were generated.

**Table 1:** Risk stratified assignment.

Risk Category	Hypertension Values and 10-year CVD Risk
Very low risk	SBP<120 and/or DBP<80 and 10-year CVD risk< 10%
Low risk	120≤SBP<140 and/or 80≤DBP<90 and 10-year CVD risk< 10%
Moderate risk	140≤SBP<160 and/or 90≤DBP<100 or 10%≤10-year CVD risk <20%
High risk	SBP≥160 and/or DBP ≥100 or 10-year CVD risk ≥20%

SBP–Systolic blood pressure; DBP–Diastolic blood pressure; CVD–Cardiovascular disease

### Model description

Our model was implemented as a directed graph in Python using the NetworkX graph library. Each node represented a possible state an individual could be assigned to and the edges represented possible transition between these states. Individuals began in an initial risk state for CVD events (very low, low, medium or high) and then were transitioned to two types of nodes: 1) acute event nodes (stroke, MI, angina, cardiac arrest) and 2) post-event nodes that could only be reached after the occurrence of an acute event. Time step of the model was 1 year with calculated yearly transition probabilities based on the 5-year risk profiles described above. Individuals in the model had multiple assigned characteristics: age, gender, risk of experiencing an acute event based on very low, low, medium, high risk groupings (proportion varied in 5-year age increments), and intervention type (no intervention or medication management with could include advice on lifestyle modifications). These characteristics along with CVD risk were used as a multi-dimensional index for transition probabilities between states. Gender was a static characteristic while age was updated every time step and risk was assigned at the end of every time step. As individuals age, they were assigned in different proportions to the risk categories; therefore, more individuals were in the high risk and moderate risk categories as they aged. These risk categories were based on the actual prevalence of risk factors in the Kenyan population as described above. Table 2 provides the disease progression parameters used in the model. An individual could remain on the same node for multiple time steps if none of the possible connected nodes were selected based on the associated transition probabilities. Once the first acute event occurred the individual entered the disease transition phase of the model. The type of first acute event (stroke, MI, angina, cardiac arrest) determined the general life history of an individual. For example, if the first acute event was a stroke, then the subsequent states included post-stroke, repeat stroke or MI event (see Table 2 for events).

**Table 2:** Disease progression parameters for the CVD microsimulation modeling.

Parameters	Values	Source
*CVD Risk*		
Assignment to CVD risk categories	Based on age, sex and risk conditions	D’Agostino et al.[15]
Stroke event risk		Wolf et al. [16]
CHD event risk		Anderson et al. [17]
*CHD Event Distribution*		
% Cardiac Arrest	0.100	Perman et al. [18]
% MI (males)	0.350	White et al. [19]
% MI (females)	0.200	White et al. [19]
% Angina	100% Minus % CHD events	
*Cardiac Arrest Outcomes*		
Acute mortality (1 year)	0.954	Nichol et al. [20]
Subsequent year mortality	0.040	Law et al. [21]
*MI Outcomes*		
Acute mortality (1 year)	0.050	Ogengo [22]
subsequent year mortality	0.040	Law et al. [21]
Repeat MI	0.064	Jokhadar et al. [23]
Mortality repeat MI	0.100	Law et al. [21]
*Angina Outcomes*		
Acute mortality (1 year)	0.045	Capewell et al. [24]
Subsequent year mortality	0.030	Law et al. [21]
Subsequent years MI	0.035	Hemingway et al. [25]
Mortality after MI	0.050	Ogengo [22]
*Stroke Outcomes*		
Acute mortality (1 year)	0.380	Mudzi et al [26]; Owalabi et al. [27]
Subsequent year mortality	0.050	Law et al. [21]
Repeat stroke event	0.040	Hardie et al. [28]
Subsequent years MI	0.022	Touze et al. [29]

A simulation run was initiated with a cohort of 1 million (equal distribution of males and females). Each individual was assigned the same start age of 25 years and the simulation generated a life history for each individual till death. As individuals age, they are assigned different risk categories based on the prevalence of risk factors by age in the Kenyan population. Therefore, we do not only look at a 25-year old but the entire trajectory as the cohort ages through the model. We decided to use a cohort model instead of the population-based model (which is a snap shot at a given time) because the risk profiles for the reduction in outcomes have to be estimated over a long period of time and our objective was to evaluate the role of risk stratified medication management over the lifespan. We estimate the effectiveness over the life course of an individual and therefore we need to model the impact for the cohort as individual ages in our model. At every time step, individuals could die of non-CVD causes or CVD events. CVD related deaths were implemented as deaths caused directly by acute CHD (MI, angina, cardiac arrest) events, acute stroke events, and those that occurred in subsequent time steps. Acute deaths were defined as those that occurred within 1 year of an event while subsequent deaths occurred after the initial one-year period (implemented as a pre-specified mortality rate at each time step). The impact of medication management was determined by adjusting the rate of acute event transition probabilities by the anticipated percentage decline in CHD and stroke events.

### Interventions, effectiveness and cost

We compared the base case of no intervention with medication management for selected risk groups: (1) high risk only, and (2) moderate and high risk. We report the outcomes for compliance with medication management at a 50% level. This level of compliance was chosen based on expert opinion provided by practicing cardiologists and primary care physicians in Kenya. A very small proportion of Kenyans, less than 5%, were on medication to manage their hypertension in 2015 [7] but there is a growing awareness especially in urban areas. Increasing this to half those in need was seen as viable by experts and would be a significant achievement; increasing it beyond this level would likely require additional interventions to improve awareness and education on risk factors of uncontrolled hypertension. The key issue in Kenya though, as in other settings, is the ability to maintain high levels of compliance over the long-term [5]. To simulate impact of long-term compliance, we evaluated different levels of effectiveness; with low levels of compliance, lower level of effectiveness will be achieved. We determined the effectiveness of medication management by applying the average decrease in CHD and stroke events based on reduction in blood pressure that were reported in meta-analysis of hypertension trials [11]. For a reduction of 10mmHG of SBP (or 5mmHG of DBP), there was an average decrease of 22% in CHD events and about 40% decrease in strokes. We used this as our best-case scenario or highest rate of reduction that could be achieved with medication management in the real-world setting (high levels of long-term compliance). We present a 11% reduction in CHD and 20% reduction in strokes as the likely average decline that can be achieved under real world conditions (moderate level of long-term compliance). In addition, for comparison we also present a scenario of worse-case scenario of a very low rate of 5% reduction in CHD and 10% reduction in strokes (low or intermittent long-term compliance). A very low level of reduction in average hypertension values could occur when the adherence over time is suboptimal. [30]

We determined disability adjusted life years (DALYs) and total cost of medical services to calculate incremental cost per DALY for the interventions. We present data for those aged 25 to 69 years to assess impact of medication management on premature mortality. We derived the DALYs based on standard methodology, without age weighting, and the disability weights were drawn from the WHO Global Burden of Disease project and reported in Table 3. [31, 32] Disability weights were assigned at each time step (1 year); acute weight was applied during the year of the event and non-acute weight in subsequent years. Cost estimates for hypertension management and acute treatments were drawn from a recent study on the cost of non-communicable diseases in Kenya. [33] Our analysis is presented from a health system perspective and therefore we include health care costs but exclude indirect costs related to productive loss. Table 3 also presents the base case as well as the low and high cost values used in the model to assess sensitivity of the incremental cost-effectiveness to changes in the cost parameters. Based on standard WHO guidance, medication management for hypertension was considered very cost-effective if the cost per DALY averted was less than the gross domestic product (GDP) per capita and cost-effective if between one and three times GDP per capita. The GDP per capita in Kenya was US$1,595 in 2017 and three times this value ($4,785) was used as the threshold for cost-effectiveness in this study. Given the budget implications of providing medications to all those at risk at the population level, we focused our analysis on identifying cost-effective scenarios. We present future costs and DALYs discounted at a 3% level.

**Table 3:** Disability and cost parameters for the CVD microsimulation modeling.

Parameters	Values			Source
	Base Case	low	high	
*Disability weights*				Murray et al. [31]
Acute MI	0.439	0.405	0.477	
Non-acute MI	0.101	0.093	0.103	
Angina (acute & non-acute)	0.124	0.105	0.141	
Acute Stroke	0.92	0.782	0.990	
Non-acute Stroke	0.266	0.228	0.295	
repeat event (MI or stroke)	0.05	0.03	0.07	
*Cost estimates*				
Medication management (per year)	$378	$76	$679	Subramanian et al. [33]
Acute events (per event)				
Cardiac Arrest	$1,593	$1,026	$2,161	
MI	$7,263	$1,996	$12,529	
Angina	$5,989	$1,237	$10,740	
Stroke	$9,292	$1,874	$16,711	
*Management after acute event (per year)*				
All CHD events	$300			Gazianio et al. [34]
Stroke	$900			

## Results

We present DALYs, cost and incremental cost per DALY gained to evaluate the cost-effectiveness of medication management for hypertension. Table 4 shows the cost-effectiveness of hypertension medication use compared to no medications using an estimated medication management cost of $378 per year. We present two intervention scenarios: (1) 50% of those classified as moderate or high risk receive medications, and (2) 50% of those classified as high risk receive medications. We show three different levels of effectiveness based on projected reductions in CHD and stroke events. For the medium level of effectiveness, the incremental cost per DALY gained ranged from $13,336 to $22,876, which are all above the $4,785 threshold. At high and low effectiveness, medication management for hypertension at this cost level is also not cost effective. See supplementary materials for assessment of stochastic uncertainly (Figures A and B in S1 File) and tornado diagram with one-way sensitivity analysis (Figures C and D in S1 File).

**Table 4:** Medication management for hypertension—DALYs, cost and incremental cost per DALY.

	Moderate and High Risk Individuals			High Risk Individuals Only		
	DALY Gained (per 100,000)	Additional Cost (per 100,000)	Incremental Cost per DALY gained	DALY Gained (per 100,000)	Additional Cost (per 100,000)	Incremental Cost per DALY gained
	(years)	(US dollars)	(US dollars)	(years)	(US dollars)	(US dollars)
High Effectiveness (Mean reduction of 10mmHG of SBP or 5mmHG of DBP)						
Women	9,016	$103,023,555	$11,427	3,441	$39,757,048	$11,554
Men	11,443	$111,056,637	$9,705	5,172	$34,630,583	$6,696
Medium Effectiveness (Mean reduction of 5mmHG of SBP or 2-3mmHG of DBP)						
Women	4,683	$107,137,377	$22,876	2,345	$41,749,455	$17,805
Men	5,974	$118,004,196	$19,753	2,868	$38,243,137	$13,336
Low Effectiveness (Mean reduction of 2-3mmHG of SBP or 1-2mmHG of DBP)						
Women	2,090	$110,041,317	$52,645	1,344	$42,819,468	$31,866
Men	2,833	$120,437,650	$42,516	1,473	$40,045,158	$27,190

**Fig 2:**
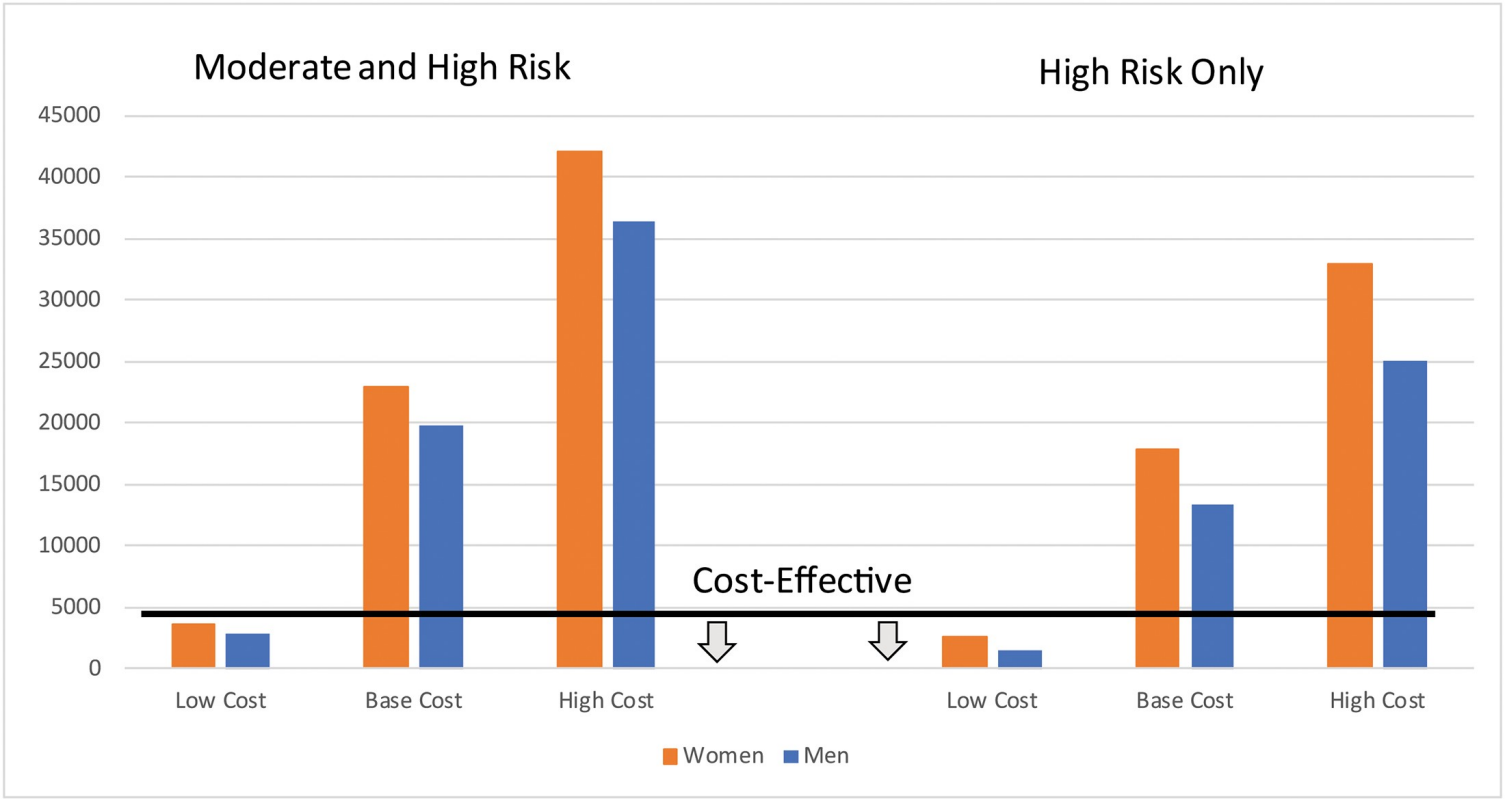
Incremental cost per DALY gained with medication management by risk group and cost.

In Fig 2, we present the changes in the incremental cost-effectiveness of medication management based on a lower ($76) and higher ($679) annual cost of hypertension medications compared to the base case cost ($378) at a medium level of effectiveness. The cost-effectiveness threshold is shown on the figure as a black line; values below the line are considered cost-effective. At only the low cost estimate was hypertension medication management a cost-effective approach in decreasing mortality and morbidity among both men and women at moderate or high risk for CVD events. Variation in cost had a large impact on the incremental cost per DALY gained even when only the high risks group were treated; the estimates ranged from $1,505 to $25,128 for men and $2,552 to $33,008 for women.

Table 5 presents the estimates under the low-cost ($76 per year medication cost) scenario for both the high and low levels of effectiveness. At the high level of effectiveness, treatments for both men and women at moderate and high risk for CVD events were cost-effective. Under low levels of effectiveness of medication management, treating high-risk men was the only cost-effective option.

**Table 5:** Incremental cost per person 25 to 70 years based on high and low effectiveness of medication management (assuming low cost of $76 per year).

	Moderate and High Risk Individuals			High Risk Individuals Only		
	**High Effectiveness (Mean reduction of 10mmHG of SBP or 5mmHG of DBP)**			**High Effectiveness (Mean reduction of 10mmHG of SBP or 5mmHG of DBP)**		
	**DALY Gained (per 100,000)**	**Additional Cost (per 100,000)**	**Incremental Cost per DALY gained**	**DALY Gained (per 100,000)**	**Additional Cost (per 100,000)**	**Incremental Cost per DALY gained**
	(years)	(US dollars)	(US dollars)	(years)	(US dollars)	(US dollars)
Women	9,016	$12,354,788	$1,370.33	3,441	$3,982,818	$1,157.43
Men	11,443	$9,936,961	$868.36	5,172	$571,888	$110.58
	**Low Effectiveness (Mean reduction of 2-3mmHG of SBP or 1-2mmHG of DBP)**			**Low Effectiveness (Mean reduction of 2-3mmHG of SBP or 1-2mmHG of DBP)**		
	**DALY Gained (per 100,000)**	**Additional Cost (per 100,000)**	**Incremental Cost per DALY gained**	**DALY Gained (per 100,000)**	**Additional Cost (per 100,000)**	**Incremental Cost per DALY gained**
	(years)	(US dollars)	(US dollars)	(years)	(US dollars)	(US dollars)
Women	2,090	$20,161,300	$9,645.36	1,344	$7,130,577	$5,306.52
Men	2,833	$20,319,408	$7,173.07	1,473	$6,166,494	$4,186.93

In Table 6, we explore the budget impact of providing hypertension medications to both the moderate and high risk subpopulations, or to just the high risk individuals based on different levels of medication cost. The cost of medications is partially offset by the decrease in treatment cost for stokes, heart attacks and other CVD events but additional resources will still be required. The costs provided in Table 5 represent the average across all individuals aged 25 to 69 years in the population. For a cohort of 100,000 men, the additional cost of implementing medication management for only the high-risk group was $4,316,000 (average cost of $43.16) with low medication cost and this increased to $72,057,000 (average cost of $720.57) with high medication cost. Cost estimates were higher for all other groups. See supplementary materials for additional details on the cost of hypertension medication management and treatment of acute events (Figures E and F in S1 File).

**Table 6:** Average cost of medication management (based on medium level effectiveness).

	Moderate and High Risk Individuals		High Risk Individuals	
	Women	Men	Women	Men
	(US dollars)	(US dollars)	(US dollars)	(US dollars)
High medication cost (reflects cost in the private sector)	1,970.79	2,179.95	773.96	720.57
Base medication cost (average medication cost)	1,071.37	1,180.04	417.49	382.43
Low medication cost (reflects cost in the public sector)	168.97	176.81	59.84	43.16

## Discussion

We developed a microsimulation model to evaluate the potential cost-effectiveness of the use of medication management to reduce the high levels of blood pressure among men and women in Kenya. Hypertension medication management for individuals at moderate and high risk of CVD events could be cost-effective but it will depend on both the level of effectiveness that can be achieved in the real-world setting as well as the cost of the hypertension medications. If an average reduction of at least 5mmHG of SBP or 2.5mmHG DBP can be achieved, representing the best-case scenario of a 11% reduction in CHD events and 20% reduction in stroke events, then an annual medication management cost of about $80 per person or lower will be cost-effectiveness. As expected, treating only those at a high risk will be more cost-effective than targeting both moderate and high risk individuals. Treating men at high risk remains cost-effective even when we considered very low level of effectiveness. Given the high prevalence of hypertension in Kenya, which affects almost a quarter of the adult population, a substantial investment will be required even at a low annual medication cost.

Similar to the findings in this analysis, a modeling study focused on the Nigerian setting found that medication management for hypertension control could be potentially cost-effective but the incremental cost effectiveness estimates were sensitive to changes in the underlying assumptions. [35] Another study assessing use of hypertension medications in South Africa found that risk stratification and treatment based on blood pressure levels alone was both more expensive and less effective than triage based on absolute risk of CVD events. [36] This study also reported that the cost of hypertension treatment was a key driver of the cost-effectiveness of the scenarios evaluated. Other studies have also highlighted the importance of accurate cost estimates and this goes beyond just the cost of hypertension medications as there are other monitoring and laboratory costs that may be associated with managing overall CVD risk. [37–39] Based on this growing body of evidence, we can conclude that the cost of the hypertension medications is a critical factor in the decision-making process.

Given the lack of empirical studies in Africa, ours and other modeling studies, cannot draw concrete conclusions on the effectiveness. Therefore, our analysis presents potential scenarios under which hypertension medication management can be cost effective and we have indicated that under almost all effectiveness scenarios, treating high-risk individuals with low-cost hypertension medication will likely be a cost-effective option. Therefore, policy makers now have information that provision of hypertension management in the public sector clinics (which reflect the low-cost estimates) for high risk patients can be a cost-effective use of funds. Further research is required to draw concrete conclusions and one of key lessons learned from the analysis presented in this manuscript is the urgent need for locally relevant studies on the risk probabilities of CVD events related to hypertension.

In this study, we focused on the role of medication management but modifiable behaviors, including cessation of tobacco use, reduction of salt in the diet, consuming fruits and vegetables, regular physical activity and avoiding harmful use of alcohol, have been shown to also reduce the risk of CVD. [40] There is lack of evidence on the level of behavior change that can be achieved so risk factors for CVDs can be reduced. Information is needed on effective approaches to foster healthy lifestyle choices which is likely to require multilevel interventions targeted at the individual, family and community. [41] Given this dearth of evidence, we have not specifically included behavior change in our modeling scenarios, but we do acknowledge that lifestyle modification is an important aspect to consider in future studies. Information on both the effectiveness as well as the cost are required to systematically compare behavior change with medication management. Long-term compliance with these approaches is also essential to project potential reduction in morbidity and mortality. Additionally, here is a need to understand the benefits of the combined effects of both medication and lifestyle modification.

The strength of this analysis is that we were able to use individual level data on hypertension as well as other risk factors from the Kenyan setting to generate the risk stratifications used in the model. Additionally, the cost of hypertension medications was based on information directly related to the experiences of the Kenyan population. In our model though, we assumed a uniform cost for hypertension management. The cost of medication management may differ across individuals and it is important to control for this variability in future analysis. Our cost estimates though are based on the mix of medications used to treat patients in Kenya and account for the proportion using one, two, three or four medications. Another key limitation is the lack of Sub-Saharan or Kenyan specific information on the risk of CVD events and mortality. There are no data-based equations to determine risk of hypertensive events to validate the model results. We applied Kenyan risk factors to risk profiles developed using largely Caucasian patient cohorts. These risk scores may not accurately reflect the underlying probability of CVD events and mortality. There may be unique factors that impact CVD risk based on age and gender profiles in Sub-Saharan Africa that we may not have captured. For example, there is some evidence to indicate that the Sub-Saharan African population may have a high risk of experiencing acute heart failure due to the presence of hypertension. [42] Large scale longitudinal cohort studies are needed to explore these issues, but this data will only be available in the distant future. In the meanwhile, future modeling studies should explore additional s consequences of hypertension in African cohorts to provide systematic evaluation that can guide policies while we await data from longitudinal studies. As in any modeling study though, long terms projections of cost and outcomes are subject to uncertainty as there could be multiple underlying factors that could change over time. Additionally, there could be variation based on underlying comorbid conditions as diabetic patients with hypertension have a much higher risk of CVD events that non-diabetes as shown in studies from other settings.[43] Risk scores based on longitudinal data from African cohorts is a key gap in the literature and there is an urgent need for this information as it will help improve model predictions and also support the implementation of evidence-based interventions that reflect the underlying population dynamics. Furthermore, our modeling was conducted from a health system perspective and we did not include indirect or productive loss. Indirect cost related to hypertension can be substantial and it will be important to consider these costs in future studies.

In conclusion, the use of comprehensive CVD risk assessment may be cost-effective in the Kenyan setting especially for the high-risk group of men and women. The results are sensitive to the cost of the medications as well as the potential effectiveness that can be achieved in terms of reduction in blood pressure and other risk factors for CVD events. Medication management requires good adherence over the long term and patient follow up studies in the Kenyan setting are required to provide guidance on the effectiveness that can be achieved in the real-world. Such studies will provide valuable input parameters that can be used in microsimulation models to develop concrete policy recommendations.

## Supporting information

S1 File Appendix. (DOCX)
