# Supplementary material for: Cost-effectiveness of risk stratified medication management for reducing premature cardiovascular mortality in Kenya
Source: PLoS One. 2019 Jun 25;14(6):e0218256. doi: 10.1371/journal.pone.0218256 (PMC6592597; doi:10.1371/journal.pone.0218256)
Supplement: S1 File — (DOCX) [file pone.0218256.s001.docx]

**Appendix**

**Generating cardiovascular risk profiles**

Using the Kenya STEPwise survey data, we derived individual level information on the prevalence of risk factors including hypertension (both SBP and DBP values), tobacco use, age, gender, diagnosis of diabetes and cholesterol levels (hyperlipidemia). We used risk estimates developed from the Framingham study to estimate the risk of CVD events for each individual. Manuscripts [15-17] based on the Framingham study, report scores for each type of risk factor which can be summed up to generate a total score for an individual. The manuscripts also provide tables that link the score to the 10-year risk of coronary heart disease (CHD) or stroke. Using these estimates, we derived 1-year probabilities and then created risk groups of very low, low, moderate and high for each 5-year age groupings. The definitions for the risk categories are provided in the manuscript (Table 1).

**Table A: Risk Group and CVD Events Probability - Males**

| **Age Group** | **Risk Group** | **Risk Group Distribution** | **1-year CHD risk** | **1-year Stroke risk** |
| --- | --- | --- | --- | --- |
| 25-29 | Very Low | 34.87% | .00000 | .00000 |
| 25-29 | Low | 54.23% | .05000 | .00000 |
| 25-29 | Moderate | 9.59% | .15667 | .25000 |
| 25-29 | High | 1.30% | .28000 | .38000 |
| 30-34 | Very Low | 28.92% | .00000 | .00000 |
| 30-34 | Low | 49.39% | .08000 | .05000 |
| 30-34 | Moderate | 17.16% | .15667 | .28000 |
| 30-34 | High | 4.53% | .28000 | .40000 |
| 35-39 | Very Low | 27.20% | .14037 | .00000 |
| 35-39 | Low | 45.19% | .21914 | .05000 |
| 35-39 | Moderate | 20.08% | .36252 | .30000 |
| 35-39 | High | 7.53% | .45368 | .45000 |
| 40-44 | Very Low | 24.75% | .26831 | .10000 |
| 40-44 | Low | 42.42% | .30561 | .15000 |
| 40-44 | Moderate | 21.72% | .50098 | .34000 |
| 40-44 | High | 11.11% | .61322 | .49000 |
| 45-49 | Very Low | 23.91% | .47959 | .18000 |
| 45-49 | Low | 36.96% | .44918 | .22000 |
| 45-49 | Moderate | 25.01% | .77697 | .38000 |
| 45-49 | High | 14.12% | .75474 | .54000 |
| 50-54 | Very Low | 18.18% | .49544 | .27905 |
| 50-54 | Low | 30.00% | .61489 | .32583 |
| 50-54 | Moderate | 35.45% | .87696 | .45764 |
| 50-54 | High | 16.36% | 1.29648 | .60227 |
| 55-59 | Very Low | 10.89% | .52705 | .29568 |
| 55-59 | Low | 18.81% | .62011 | .35849 |
| 55-59 | Moderate | 37.66% | 1.03820 | .50812 |
| 55-59 | High | 32.63% | 1.34947 | .76551 |
| 60-64 | Very Low | 8.51% | .69270 | .35913 |
| 60-64 | Low | 2.13% | .92000 | .40000 |
| 60-64 | Moderate | 54.26% | 1.12580 | .53746 |
| 60-64 | High | 35.11% | 1.51809 | .84966 |
| 65-69 | Very Low | 4.62% | .89001 | .47473 |
| 65-69 | Low | 1.54% | 1.10000 | 0.5500 |
| 65-69 | Moderate | 38.46% | 1.53686 | .71322 |
| 65-69 | High | 55.38% | 1.98516 | 1.28540 |
| 70+ | Very Low | 4.62% | .98289 | .52802 |
| 70+ | Low | 1.54% | 1.20000 | .58000 |
| 70+ | Moderate | 38.46% | 1.64154 | .72120 |
| 70+ | High | 55.38% | 2.19620 | 1.37007 |
|  |  |  |  |  |

**Table B: Risk Group and CVD Events Probability – Females**

| **Age Group** | **Risk Group** | **Risk Group Distribution** | **1 -year CHD risk** | **1-year Stroke risk** |
| --- | --- | --- | --- | --- |
| 25-29 | Very Low | 60.37% | .00000 | .00000 |
| 25-29 | Low | 33.49% | .03000 | .00000 |
| 25-29 | Moderate | 4.92% | .10000 | .13317 |
| 25-29 | High | 1.21% | .15000 | .24000 |
| 30-34 | Very Low | 45.01% | .00000 | .00000 |
| 30-34 | Low | 42.50% | .05000 | .03000 |
| 30-34 | Moderate | 9.52% | .10000 | .16317 |
| 30-34 | High | 2.97% | .15000 | .29000 |
| 35-39 | Very Low | 31.74% | .08000 | .00000 |
| 35-39 | Low | 41.62% | .10000 | .03000 |
| 35-39 | Moderate | 17.66% | .15000 | .19931 |
| 35-39 | High | 8.98% | .20000 | .33000 |
| 40-44 | Very Low | 25.79% | .14230 | .05000 |
| 40-44 | Low | 45.24% | .18872 | .10000 |
| 40-44 | Moderate | 18.65% | .26667 | .21316 |
| 40-44 | High | 10.32% | .37210 | .38000 |
| 45-49 | Very Low | 20.83% | .18655 | .11766 |
| 45-49 | Low | 37.50% | .26854 | .16171 |
| 45-49 | Moderate | 22.92% | .44944 | .23560 |
| 45-49 | High | 18.75% | .42425 | .44975 |
| 50-54 | Very Low | 14.97% | .28527 | .11937 |
| 50-54 | Low | 34.13% | .39386 | .16281 |
| 50-54 | Moderate | 29.34% | .68645 | .28316 |
| 50-54 | High | 21.56% | .74810 | .48149 |
| 55-59 | Very Low | 18.42% | .36698 | .13331 |
| 55-59 | Low | 30.26% | .51375 | .20811 |
| 55-59 | Moderate | 28.95% | .74327 | .29526 |
| 55-59 | High | 22.37% | .80703 | .50152 |
| 60-64 | Very Low | 19.08% | .43455 | .17927 |
| 60-64 | Low | 21.37% | .51438 | .23966 |
| 60-64 | Moderate | 32.06% | .78809 | .38534 |
| 60-64 | High | 27.48% | 1.01169 | .64974 |
| 65-69 | Very Low | 9.17% | .51546 | .23265 |
| 65-69 | Low | 18.33% | .60000 | .35083 |
| 65-69 | Moderate | 34.17% | .86636 | .52582 |
| 65-69 | High | 38.33% | 1.20000 | 1.05455 |
| 70+ | Very Low | 9.17% | .55861 | .41291 |
| 70+ | Low | 18.33% | .63000 | .63209 |
| 70+ | Moderate | 34.17% | .89943 | .94094 |
| 70+ | High | 38.33% | 1.25697 | 1.89115 |
|  |  |  |  |  |
|  |  |  |  |  |

**Uncertainty Analysis and Incremental Cost-Effectiveness Ratio (ICER)**

Figures A and B present the distribution of the effectiveness (disability adjusted life years gained – DALY gained) and cost and the resulting ICER in the cost-effectiveness plane. These are based on 50 runs with one million individuals each and the estimates reported are for a cohort of 100,000. Two separate figures are presented for men and women for scenarios including both moderate and high risk individuals, under the medium level of effectiveness (mean reduction of 5mmHG of SBP or 2-3mmHG of DBP), with the base case annual hypertension medication cost of $378 a year. The plots show that there is a large variation in the effectiveness measure as shown in the range of the DALYs presented. On the other hand, the cost differences are less variable, with a range of about $3 to $4 million for the 100,000 individuals reported in the graphics. This indicates that there is some stochastic variation in the model but overall even with this variation the results presented in Table 4 (see manuscript) are generally stable. For example, in Table 4 we report $19,753 as the ICER for men (medium effectiveness, high and moderate risk). Using the average of the estimates from the figure below, the ICERs could be $22,673 ($117,859,108/5,198) with a range from about $16,000 to $29,000. Therefore, the conclusions drawn from Table 4 do not change but these findings indicate that values close to the cost-effectiveness threshold may need to be further investigated due to the inherent variation in the model estimation.

**Figure A: Moderate and High Risk Men (100,000 individuals)**


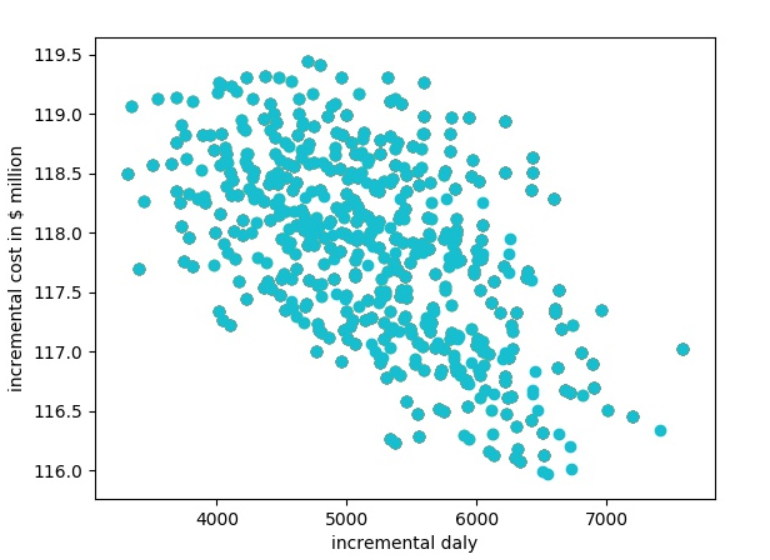


**Figure B: Moderate and High Risk Women (100,000 individuals)**


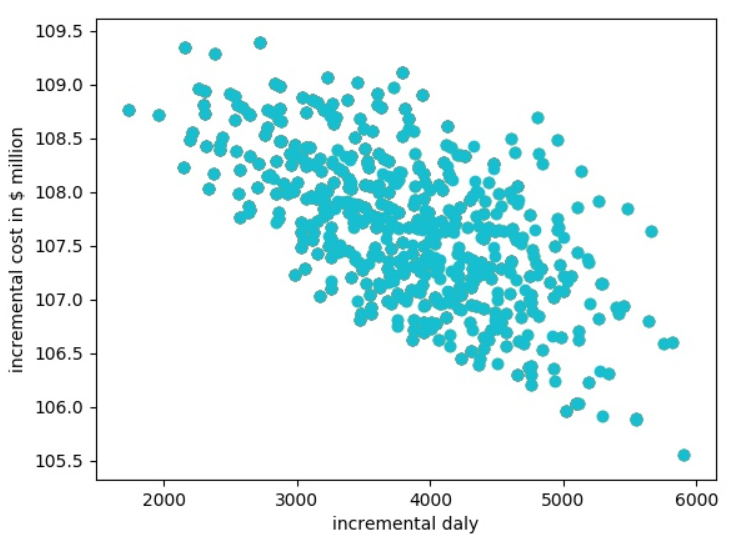


**One-way Sensitivity Analysis**

We present the one-way sensitivity analysis for men and women separately in the tornado diagrams below. The high-low estimates used in the sensitivity analysis for cost, effectiveness of hypertension management and disability weights are provided in the manuscript. We used 10% lower and 10% higher values for CVD risk and mortality estimates. Cost remains the key parameters with the largest range in incremental cost per DALY (low to high estimates of cost).

**Figure C: Sensitivity Analysis - Men**

**Figure D: Sensitivity Analysis - Women**

**Cost Estimates Generated from the Modeling Runs**

In the figures below, we provide the costs for the hypertension medication, acute treatments and post events. The costs shown are the actual cost for the cohort and not the incremental cost. It is clear that the cost of medications is lower for the high risk group (Figure E) as this is a much smaller cohort of individuals than the cohort that includes both high risk and moderate risk individuals (Figure F).

**Figure E: Cost for High Risk Only (100,000 individuals; $378 medication cost per year)**

**Figure F: Cost for High and Moderate Risk (100,000 individuals; $378 medication cost per year)**
